# Diseases of the Colon and Their Surgical Treatment

**Published:** 1911-01

**Authors:** 


**Diseases of the Colon and their Surgical Treatment.** By **P. Lockhart Mummery,** F.R.C.S., Eng., Jacksonian Prizeman and late Hunterian Professor, Royal College of Surgeons, London. Small octavo, pp. 330. Illustrated. New York: William Wood & Co. 1910. (Cloth, $3.25.)

Founded upon the thesis which was awarded the Jacksonian prize by the Royal College of Surgeons we have in this work a practical textbook that takes up, and makes clear, much that heretofore has been but imperfectly understood. In the twenty-two chapters of his book, the author deals in a most comprehensive manner with the various morbid conditions that are likely to be met with in practice. The colon is a very inaccessible portion of the human body and when the site of the disease is unknown it is not easy to correctly diagnosticate the condition or to ascertain the position and nature of the lesion. In chapter V every means that will aid in an understanding of diseased conditions is discussed. The proctologist, and that term includes nearly every general practitioner of medicine, will have need for this book.

